# A strategy for successful dual‐species protein expression of genes with non‐optimal codon usage destined for bacterial and yeast cell factories

**DOI:** 10.1002/btpr.3482

**Published:** 2024-05-17

**Authors:** Marcus Wäneskog, Trine Bertram Rasmussen, Emil D. Jensen

**Affiliations:** ^1^ Novo Nordisk Foundation Center for Biosustainability Technical University of Denmark Kgs. Lyngby Denmark

**Keywords:** bacteria, codon optimization, inducible expression, mNeonGreen, tRNA, yeast

## Abstract

Recombinant protein expression on an industrial scale traditionally utilizes one of two microbial workhorses: *Escherichia coli* or *Saccharomyces cerevisiae*. Additionally, random protein engineering of enzymes and proteins aimed for expression in *S. cerevisiae* are often mutagenized and pre‐screened in *E. coli* before expression in yeast. This introduces artificial bottlenecks as the bacterial expression vector needs to be substituted for a yeast expression vector via sub‐cloning, and the new library re‐evaluated before a final screening in yeast. Here, we put forward a protein expression and engineering strategy that involves the use of a dual‐host shuttle vector (pYB‐Dual) designed with both a strong inducible yeast promoter (pGAL1), and a strong inducible bacterial promoter (pT7‐RNAP), which allows for inducible protein expression in both species. Additionally, we demonstrate that by transforming the pYB‐Dual vector into the *E. coli* strain Rosetta 2, which has elevated levels of 7 rare tRNAs, we can achieve high‐level protein expression in both yeast and bacteria, even when using a mNeonGreen gene codon optimized for yeast. This dual expression vector is expected to remove bottlenecks during protein engineering of commercially important enzymes destined for high‐titer expression in yeast.

## 1 INTRODUCTION

Recombinant protein expression and large‐scale purification are cornerstones of the biotech industry. Such heterologous proteins are often expressed by either a genetically engineered protein production strain of *Escherichia coli* or a fed‐batch‐fermentation stable strain of *Saccharomyces cerevisiae*.^1^, ^2^ Human insulin is perhaps the most famous example of a high‐value peptide expressed recombinantly from a microorganism. Insulin was initially produced at high titers from *E. coli*,^3^ while today *S. cerevisiae* is the most common production host.^1^, ^2^ This transition from recombinant peptide expression in bacteria to yeast is not difficult to understand. Compared to bacteria, yeast has several significant advantages as a cell factory. Yeast supports stable fed‐batch fermentation at high‐cell densities, and most recombinant proteins and peptides can easily be secreted into the culture media for continuous collection and purification.^1^, ^2^ However, bacteria are easier to engineer and better suited for the screening of large libraries of protein variants.

Different approaches have previously aimed to bridge the divide between bacterial and yeast protein expression. Yuan et al.^4^ discovered that the strong bidirectional yeast promoter, pGAL1/10, is also a weak transcriptional start site in *E. coli*. They also reported that this yeast promoter was subject to a minor catabolite repression in *E. coli*. with a <2‐fold differential expression when the bacteria were grown in media lacking glucose. While this discovery allows for easy dual‐species expression in both bacteria and yeast from the same promoter construct, it does not allow for a high‐titer expression or precise control of protein expression in bacteria. Saida et al.^5^ on the other hand designed a dual species expression vector able to express a fragment of the S1 ribosomal protein in both *E. coli* and *S. cerevisiae* from a construct containing both a strong inducible yeast promoter (pGAL1) and a strong titratable bacterial promoter (pT7‐RNAP). Although, while translation was optimized for bacteria by the use of a strong Shine‐Dalgarno sequence, the translation initiation and protein expression was comparably weaker in yeast, as the authors used the original Kozak consensus sequence identified in 1986.^5^, ^6^ The original Kozak sequence (ACCATGG) has since been shown to be sub‐optimal in *S. cerevisiae*, as efficient translational initiation in yeast requires a sequence comprised almost entirely of adenines immediately upstream of the start codon.^7^ Moreover, neither Yuan et al. nor Saida et al. investigated the effect that differential codon usage has on the efficiency of protein expression in *E. coli* compared to *S. cerevisiae*, when using their dual‐species protein expression vectors.

Here we describe a dual‐host expression vector engineered to express any library, or gene‐of‐interest, from two separate inducible promoters, pGAL1 or pT7‐RNAP. Each promoter allows for high‐level transcriptional expression in either yeast (*S. cerevisiae*) or bacteria (*E. coli*). This dual‐species expression vector was further modified by providing the gene‐of‐interest with a strong bacterial ribosomal binding‐site (Shine‐Dalgarno), semi‐overlapped by a strong yeast translational enhancing sequence, which allows for strong and efficient protein translation in both yeast and bacteria. We also demonstrate that by using the *E. coli* strain Rosetta 2, which has elevated levels of 7 rare tRNAs, we can achieve an efficient protein expression of a yeast codon optimized gene‐of‐interest (mNeonGreen) in both yeast and bacteria. Using the strategy described here we propose that a large library of protein variants could first be screened and evaluated in *E. coli*, then shuttled into *S. cerevisiae* for a final evaluation before high‐titer protein expression and purification, without needing to re‐clone and re‐evaluate the new library. This strategy should thus both save time and facilitate faster innovation.

## 2 MATERIALS AND METHODS

### 2.1 Strains, DNA constructs, and growth conditions

The bacterial and yeast strains, plasmid constructs and oligos used in this study are listed in Tables S1–S3 and Figures S1 and S2 (available in the online version of this article). The yeast codon optimized mNeonGreen (mNG) was designed and synthesized by GeneStrings (Thermo Fisher Scientific). The pYB‐Dual (mNG (yeast‐opt)) vector have been deposited in Addgene (ID 216727). Bacterial strains were grown at 37°C and with shaking at 200 rpm in Lysogeny broth (LB): 10 g/L tryptone, 5 g/L yeast extract, and 10 g/L NaCl. Media was supplemented with ampicillin, or Carbenicillin, at 100 mg/L, and Chloramphenicol, at 10 mg/L. Isopropyl‐β‐d‐thiogalactopyranoside (IPTG) was added when stated. Yeast strains were grown at 30°C and with shaking at 200 rpm in synthetic drop‐out medium, minus leucine (SC‐Leu), (Sigma‐Aldrich, #Y1376). Media was supplemented with either glucose, sucrose, raffinose or galactose, as stated.

### 2.2 Bacterial mNG induction assay

Bacterial strains (Figure S1) were grown overnight in LB before diluted 1:20 in liquid LB media and incubated until OD600 = 0.6–0.7 was reached, IPTG was then added, and cells were grown for 16 h before fluorescence was measured.

### 2.3 Yeast mNG induction assay

Yeast strains (Figure S2) were grown overnight in SC‐Leu with 2% glucose before diluted 1:50 in liquid SC‐Leu media supplemented with either 2% glucose, sucrose, raffinose or galactose, or a combination of galactose and glucose, sucrose or raffinose. Cells were grown for 16 h before fluorescence was measured.

### 2.4 mNG fluorescent measurements

Bacterial or yeast samples were analyzed by a BioTek SynergyMx plate reader, with mNG excitation at 500 nm, and emission at 520 nm, with OD600 of bacterial and yeast cells measured at 600 nm. Statistical significance was calculated using the Two‐way ANOVA test (*n* = 3–4).

## 3 RESULTS

### 3.1 Gene‐of‐interest and codon optimization

**FIGURE 1:**
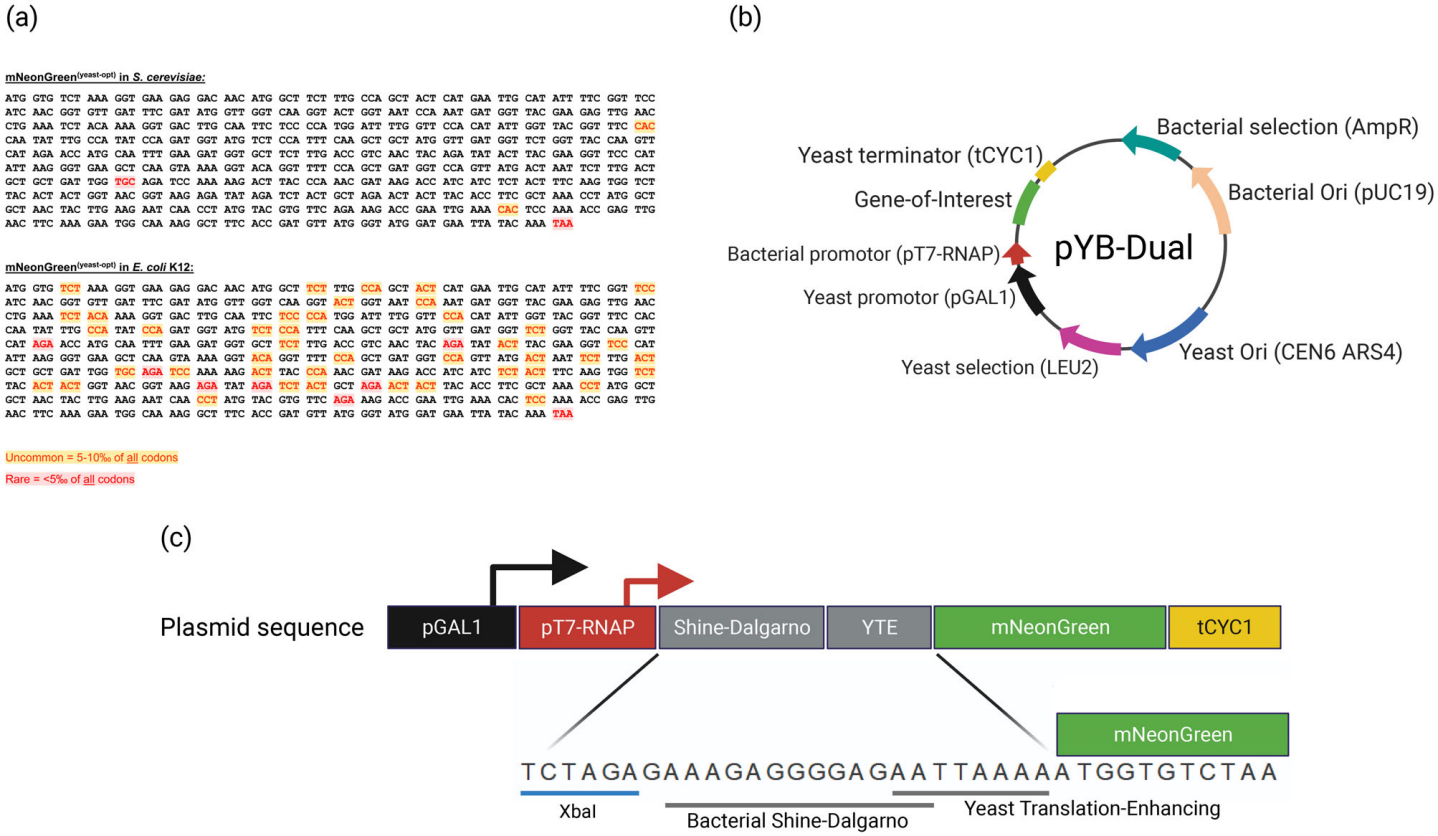
(a) Sequence of the yeast codon‐optimized mNG gene, cloned into the pYB‐Dual plasmid. Uncommon and rare codons, for when mNG is expressed in either *S. cerevisiae* or *E. coli*, were calculated using ATGme^9^ and highlighted yellow (uncommon) or red (rare) according to the threshold; 5‰–10‰ of all codons for uncommon and <5‰ of all codons for rare. (b) Schematic illustration of the pYB‐Dual plasmid. (c) The genetic arrangement of the mNG expression cassette. Illustration not to scale. Illustration created with BioRender.com.

To validate our **Y**east and **B**acteria **Dual**‐species expression vector (pYB‐Dual), we choose to express the green fluorescent protein mNeonGreen (mNG) in both the yeast *S. cerevisiae* and the Gram‐negative bacterium *E. coli*. This fluorescent protein was chosen as it is one of the brightest monomeric fluorescent proteins known to exist and therefore was expected to be easily detected in both the yeast and the bacteria, even when expressed at a low level.^8^ This consideration was important as we were uncertain of the mNG expression level we could achieve in either species with our dual‐species expression vector. Moreover, to better replicate the circumstances that normally would occur when shuttling non‐endogenous genes to a yeast cell factory, we also codon‐optimized the mNG open‐reading frame (ORF) for the end‐host: *S. cerevisiae*. This optimization removed rare codons, but also uneven GC‐content and other difficult sequence motifs, such as repeats and palindromes. This, however, meant that one rare and two uncommon yeast codons remained after the optimization (Thermo Scientific Gene Strings) (Figure 1a). If expressed in *E. coli*, this yeast optimized sequence was predicted to contain seven rare and 42 uncommon *E. coli* codons,^9^ including a very problematic region between position 525–546, containing three rare codons clustered closely together (Figure 1a).

### 3.2 Design of pYB‐Dual vector

The design of our dual‐species expression vector (pYB‐Dual) was inspired by Saida et al.,^5^ and we chose to also use the strong galactose inducible yeast promoter GAL1, together with the strong T7‐RNAP promoter. This genetic arrangement means that the entire pT7‐RNAP sequence is included into the 5′UTR of the yeast mRNA expressed from pGAL1. However, as pT7‐RNAP does not contain any ATG start codons, this sequence should have minimal effect on the proper cap‐dependent translation initiation in *S. cerevisiae* (Figure 1b). To ensure strong translational initiation in both *E. coli* and *S. cerevisiae*, we also included a semi‐overlapping bacterial and yeast translational enhancing sequence immediately upstream of the mNG gene (Figure 1c). This sequence was inspired by the original Shine‐Dalgarno sequence for strong bacterial translation initiation,^10^ and the newly identified adenine rich‐sequence for strong translational initiation in yeast.^7^ A yeast transcriptional terminator (tCYC1) was also placed downstream of the mNG ORF to ensure proper mRNA maturation in yeast. Because transcription and translation in bacteria are coupled, there is no strict requirement to include a transcriptional terminator for any synthetic gene expression in bacteria.^11^ We therefore omitted adding a bacterial terminator in our vector construct. For our plasmid backbone we chose to use a centromeric yeast origin of replication with a leucine prototrophic marker (pRS415).^12^ This pRS415 vector backbone also contains an *E. coli* pUC19 origin of replication and an ampicillin resistance gene, allowing for dual species propagation and selection.

### 3.3 Protein expression in yeast

**FIGURE 2:**
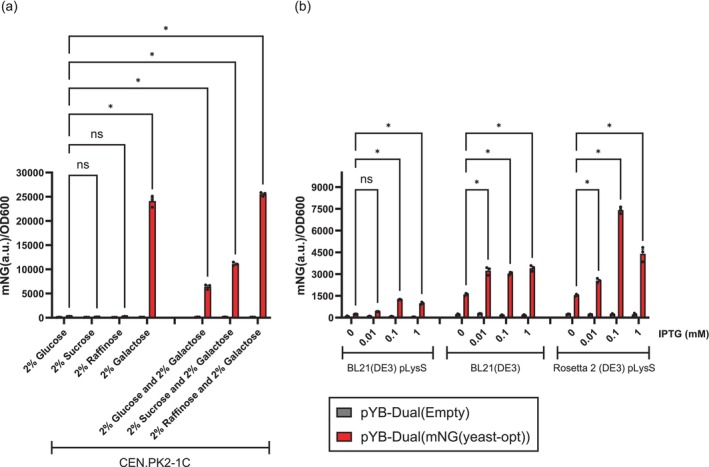
(a) mNG expression from the pYB‐Dual vector when transformed into *S. cerevisiae*, grown in synthetic drop‐out medium, minus leucine, broth (SC‐Leu). Induction from pGAL1 was achieved by 2% galactose or a combination of either 2% galactose with 2% glucose, sucrose or raffinose (*n* = 3). (b) mNG expression from the pYB‐Dual vector when transformed into *E. coli*, grown in lysogeny broth (LB). Induction from pT7‐RNAP was achieved by T7‐RNAP after IPTG induction of the lacUV5 promoter, which controls the expression of the T7‐RNAP from the DE3 prophage (*n* = 3). Statistical significance was calculated by Two‐way ANOVA with ns: *p* > 0.05 and *p ≤ 0.0001.

Next, we transformed our dual‐expression vector into *S. cerevisiae* and observed a strong mNG specific fluorescent signal after we induced expression with galactose, galactose together with raffinose, or to a lesser extent, galactose together with sucrose or glucose (Figure 2a). The mNG specific fluorescent signal was 90‐fold higher when induced by either galactose alone, or galactose and raffinose together (Table 1). With a 41‐fold fluorescent change measured for galactose and sucrose, and a 17‐fold fluorescent change measured for galactose together with glucose (Table 1). While glucose acts as a repressor of pGAL1, it should be noted that we measured yeast fluorescence in stationary phase where there is a strong possibility that most of the glucose have been depleted and that galactose is the sole carbon source remaining in the growth media. No significant mNG specific fluorescence was detected when yeast cells were grown in the absence of galactose, with either glucose, sucrose or raffinose as the primary carbon source. From these results we concluded that our pYB‐Dual plasmid construct allowed for a strong, efficient, and tight protein expression control in yeast.

**TABLE 1:** Fold‐change calculations of mNG expression above background in *S. cerevisiae*, by normalizing to the empty vector control of each sugar source, and fold‐change calculations of mNG induction, normalizing to 2% glucose.

Carbon source	*S. cerevisiae* CEN.PK2‐1C	
	Fold‐change over background (empty vector)	Fold‐change during induction (galactose)
2% Glucose	3.1	1.0
2% Sucrose	1.6	0.5
2% Raffinose	3.3	0.9
2% Galactose	201.7	89.7
2% Glucose and 2% Galactose	48.6	16.8
2% Sucrose and 2% Galactose	89.6	41.3
2% Raffinose and 2% Galactose	209.6	95.0

### 3.4 Protein expression in bacteria

To investigate the performance of our dual‐species protein expression vector in *E. coli*, we transformed the BL21(DE3) pLysS strain with our pYB‐Dual vector. This strain has the lambda DE3 prophage integrated in the chromosome, which contains the T7 RNA polymerase (T7‐RNAP) gene required for transcriptional expression from the T7‐RNAP promoter in pYB‐Dual. In BL21(DE3) pLysS, the T7‐RNAP gene is controlled by the lacUV5 promoter, which allows for titratable gene expression when induced by the lactose analog isopropyl‐β‐D‐thiogalactopyranoside (IPTG). This strain also contains the pLysS plasmid, which constitutively expresses the T7 lysozyme that inhibits the activity of the T7‐RNAP. This lowers the basal‐level activity of T7‐RNAP, allowing for tighter control of protein expression. We performed an IPTG titration experiment and measured the mNG specific fluorescent signal in BL21(DE3) pLysS, transformed with pYB‐Dual, and could observe a weak‐to‐medium fluorescent signal, compared to what we previously measured in *S. cerevisiae*. As Yuan et al. have previously demonstrated that pGAL1 is also a weak transcriptional start site in *E. coli* we were not surprised to detect a slight leaky mNG expression even in the absence of IPTG (Figure 2b). However, we did observe a 5‐fold IPTG‐dependent mNG differential expression, clearly demonstrating that the pYB‐Dual vector offers a dynamic protein expression control in *E. coli*. As we only observed a very modest fluorescent signal in *E. coli*, approximately 15‐fold lower than in *S. cerevisiae*, we were curious to investigate if this was in any way due to the inhibitory effect of the T7 lysozyme expressed from the pLysS plasmid. Toward that end, we cured our BL21(DE3) strain from the pLysS plasmid by continuously re‐streaking the strain in the absence of antibiotic selection until a plasmid free BL21(DE3) strain could be identified. This strain was then transformed with our pYB‐Dual vector, and the IPTG‐dependent mNG expression was measured. BL21(DE3) cells lacking the pLysS plasmid had a higher‐level expression of mNG regardless of the IPTG concentration (Figure 2b). However, as the basal level expression (0 mM IPTG) of mNG was higher than in the BL21(DE3) pLysS strain, we only observed a 2‐fold IPTG‐dependent induction of mNG (Table 2). Thus, we observed a higher overall expression of mNG in BL21(DE3), at the expense of dynamic range. The maximum level of mNG expression was also reached at a lower concentration of IPTG (0.01 mM), indicating that the BL21(DE3) cells have a difficult time producing large quantities of mNG protein. To investigate if this was because the mNG ORF contained numerous rare and non‐optimal *E. coli* codons, we repeated our previous IPTG titration experiments, but with the pYB‐Dual plasmid transformed into the Rosetta 2 (DE3) pLysS *E. coli* strain. This strain has elevated levels of 7 rare tRNA molecules due to extra copies of these tRNA genes inserted into the pLysS plasmid. Consequently, this strain should have an improved expression of any ORF containing many rare codons. When we measured the IPTG‐dependent expression of mNG in Rosetta 2 (DE3) pLysS we observed increased mNG specific fluorescence, compared to the BL21(DE3) pLysS strain, across all IPTG concentrations tested. Both strains also had very similar dynamic ranges (0 vs. 0.1 mM IPTG) of approximately 5‐fold (Table 2). Unfortunately, we could not repeat the BL21(DE3) pLysS versus BL21(DE3) comparison with the Rosetta 2 strains, as the extra copies of rare tRNA molecules are located on the pLysS plasmid and thus cannot be separated from the inhibitory effect of the T7 lysozyme, also located on the pLysS plasmid. Collectively, this demonstrates that our dual‐species expression vector (pYB‐Dual) can successfully express a yeast codon‐optimized gene‐of‐interest at an easily detectible level of 32‐fold above background fluorescence in *E. coli*, if the strain has an elevated level of rare tRNA molecules (Table 2).

**TABLE 2:** Fold‐change calculations of mNG expression above background in *E. coli*, by normalizing to the empty vector control for each concentration of IPTG, and fold‐change calculations of mNG induction, normalizing to 0 mM IPTG.

IPTG (mM)	*E. coli* BL21 (DE3) pLysS		*E. coli* BL21 (DE3)		*E. coli* Rosetta 2 (DE3) pLysS	
	Fold‐change over background (empty vector)	Fold‐change during induction (IPTG)	Fold‐change over background (empty vector)	Fold‐change during induction (IPTG)	Fold‐change over background (empty vector)	Fold‐change during induction (IPTG)
0	3.1	1.0	7.5	1.0	6.6	1.0
0.01	5.0	1.7	12.0	2.0	12.1	1.7
0.1	16.7	5.2	19.8	1.9	32.6	4.8
1	25.7	4.1	21.3	2.1	20.2	2.9

### 3.5 Benchmarking pYB‐Dual against conventional yeast and bacterial expression vectors

**FIGURE 3:**
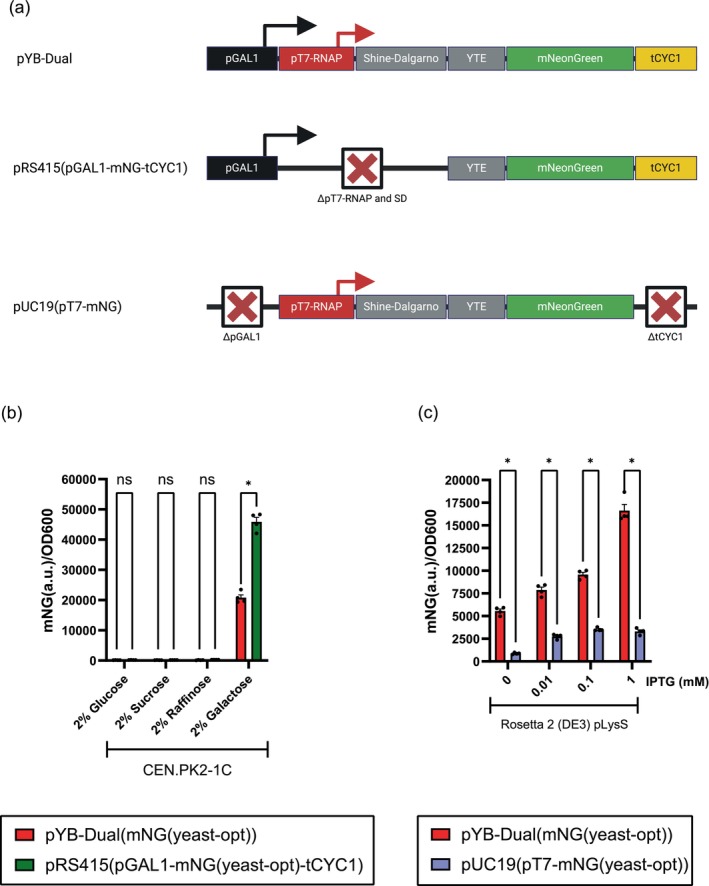
(a) Schematic illustration of the genetic arrangement of the mNG expression construct located on the pYB‐Dual plasmid, pRS415, and pUC19 plasmids. Illustration not to scale. (b) mNG expression from the pYB‐Dual and pRS415 vectors when transformed into *S. cerevisiae*, grown in synthetic drop‐out medium, minus leucine, broth (SC‐Leu). Induction from pGAL1 was achieved by 2% galactose or 2% glucose, sucrose or raffinose (*n* = 4). (c) mNG expression from the pYB‐Dual (slow growing cells) or pUC19 vector when transformed into *E. coli* Rosetta 2 (DE3) pLysS cells, grown in lysogeny broth (LB). Induction from pT7‐RNAP was achieved by T7‐RNAP after IPTG induction of the lacUV5 promoter, which controls the expression of the T7‐RNAP from the DE3 prophage (*n* = 4). Statistical significance was calculated by Two‐way ANOVA with ns: *p* > 0.05 and *p ≤ 0.0001. Illustration not to scale. Illustration created with BioRender.com.

The pYB‐Dual expression vector we describe here allows for a strong mNG protein expression in both *S. cerevisiae* and *E. coli*. However, we cannot rule out that when we change the 5′ UTR sequence of the yeast mRNA, by adding the sequence of the pT7‐RNAP promoter and bacterial Shine‐Dalgarno, we do not also alter expression efficiency in *S. cerevisiae*. Thus, to investigate this, we removed both sequences from our pYB‐Dual vector and reverted it back to a conventional yeast expression vector (pRS415) (Figure 3a).^12^ When we expressed mNG from this construct we observed approximately double the mNG expression as compared to the pYB‐Dual construct (Figure 3b). This result demonstrates that the pT7‐RNAP and Shine‐Dalgarno sequences, present in the pYB‐Dual vector, attenuates translation when part of the 5′ UTR of a yeast mRNA. Yet, even with this attenuation, the pYB‐Dual expression vector still achieves a strong mNG expression in *S. cerevisiae* (Figure 3b). Because of these findings, we also compared the expression efficiency of the pYB‐Dual vector compared to a conventional pUC19 vector in *E. coli* (Figure 3a). However, when we grow our pYB‐Dual and pUC19 transformed Rosetta 2 (DE3) pLysS strains in LB media, prior to IPTG induction, we observed that the pYB‐Dual transformed cells always required more than double the incubation time before reaching an OD600 of 0.6–0.7. This indicated to us that the low‐level leaky expression of the yeast codon‐optimized mNG protein from the pGAL1 promoter had a noticeable fitness cost in *E. coli*. To test this hypothesis, we re‐transformed the pYB‐Dual plasmid into our Rosetta 2 (DE3) pLysS strain and randomly chose 4 transformed, but slow‐growing bacterial colonies for our IPTG titration assay. We reasoned that a higher fitness burden, that is, slow growth, corresponded to higher expression of mNG. When we induced mNG expression by addition of IPTG in the slow‐growing Rosetta 2 transformants, we could observe a maximum expression >2‐fold higher than in our previous IPTG titration assay (Figures 2b and 3c). Moreover, we previously observed that normal growing pYB‐Dual transformed Rosetta 2 colonies had a maximum mNG expression at 0.1 mM IPTG (Figure 2b). While the slow‐growing pYB‐Dual transformed Rosetta 2 colonies, instead had a maximum expression at 1 mM IPTG (Figure 3c). Thus, both maximum expression and induction dynamics differed significantly between the transformed Rosetta 2 cells with a fast or slow growing phenotype. We also observed that the basal level expression of mNG, coming from the pGAL1 promoter, in the absence of IPTG was >3‐fold higher for the slow‐growing colonies compared to the normal growing colonies (Figures 2b and 3c). These observations further support our conclusion that the low‐level basal expression of the yeast codon‐optimized mNG protein causes a fitness burden to the Rosetta 2 cells.

For the pUC19 transformed cells we could not detect any significant growth defects, and thus only chose 4 randomly transformed colonies. However, unlike the pYB‐Dual versus pRS415 mNG expression in yeast, we observed that removal of the pGAL1 yeast promoter, the tCYC1 yeast terminator, the yeast origin of replication and the yeast selection marker (pUC19 (pT7‐mNG)) resulted in an >5‐fold lower level of mNG expression in *E. coli*, compared to the pYB‐Dual plasmid (Figure 3c). This >5‐fold attenuation of expression was present with or without IPTG induction (Figure 3c). Suggesting that mNG expression from the pYB‐Dual plasmid in *E. coli* is the combined result of all promoters that drive mNG transcription, that is, both pT7‐RNAP and pGAL1 (Figure 3c).

## 4 DISCUSSION

Here, we put forward a dual‐species protein expression and engineering strategy that involves the use of a dual‐host shuttle and expression vector (pYB‐Dual) optimized for high level protein expression of a gene‐of‐interest in both yeast and bacteria. We also demonstrate a possible mitigation strategy that can be implemented together with the pYB‐Dual vector, involving the over‐expression of rare tRNA molecules in *E. coli*, which can increase the efficiency of non‐endogenous protein expression. This removes a common protein expression bottleneck when expressing a protein outside of the native host. Moreover, we demonstrate the importance of selecting the right transformants before a protein expression assay. With slowing growing colonies being more capable of high‐level expression, at the cost of a significantly reduced fitness. The vector design presented here ensures high‐level of mNG expression in both yeast and bacteria by exploiting the fact that the minimal T7‐RNAP promoter sequence is free of any ATG start codons and thus can easily be incorporated into the 5′ UTR of a yeast mRNA. Translation initiation is likewise enhanced in both species by combining both a bacterial and a yeast translational enhancement sequence immediately upstream of the gene‐of‐interest. These two sequences can co‐exist upstream of the gene‐of‐interest, because the bacterial Shine‐Dalgarno is optimally located approximately at position −7 to −20, relative to the ATG, and is AG‐rich, while the yeast translation enhancement sequence is the first 8 nt immediately upstream of the ORF, and is A‐rich.^7^, ^10^ Consequently, these two sequences can easily be designed to semi‐overlap (Figure 1c). However, merging two different promoter sequences is often a precarious endeavor. For the two promoters described here, pGAL1 and pT7‐RNAP, we could observe that in yeast the pGAL1 promoter functioned better alone than together with pT7‐RNAP (Figure 3b). Nevertheless, this attenuation of expression was rather minor, with less than a 2‐fold decrease in fluorescence (Figure 3b,c). Additionally, in *E. coli*, the presence of the pGAL1 promoter upstream of the pT7‐RNAP promoter only enhanced expression, presumably through a cumulative promoter effect (Figure 3b). Thus, while the dual‐promoter construct described here is not always the most optimal approach, the potential utility that the pYB‐Dual have when shuttling genes from yeast to bacteria, or vice‐versa, more than compensates for the attenuation of protein expression in yeast. Thus, as long as a maximum protein expression in yeast in not required the pYB‐Dual plasmids should always be considered for any dual‐species protein expression experiment going forward.

Another important aspect to consider is the modularity of the dual‐expression construct presented here. The pT7‐RNAP, Shine‐Dalgarno and Yeast Translation‐Enhancing sequence can all coexist within the 5′ UTR of a yeast mRNA and mediate a high‐level of cap‐dependent translational initiation in yeast. This implies that the yeast promoter used in this study (pGAL1) could easily be replaced with any promoter of choice. This is important, considering that galactose would not always be the best choice for protein induction for a yeast cell‐factory. We therefore suggest that the construct presented here should be maintained as is, apart from the yeast promoter (pGAL1), which should be replaced with an alternative promoter of choice.

Yeast cell factories have many benefits compared to bacterial cell factories, including the lower risk‐class associated with yeast work, as *S. cerevisiae* is a non‐pathogenic organism generally recognized as safe (GRAS) by the food and drug administration (FDA) of the United States of America (USA). Moreover, fed‐batch‐fermentation stable yeast strains can easily be cultured to high cell densities, and the yeast can be engineered to secrete many different expression products such as proteins and peptides into the culture media, allowing for easy collection and purification. However, bacterial DNA transformation efficiencies are often 2–3 orders of magnitude higher than for yeast, often as high as 10^10^ transformants per μg DNA transformant.^13^ Thus, yeast becomes a limiting factor when screening large libraries of enzyme‐ and/or protein variants. To overcome this problem, we suggest that a two species screening regime can be performed using the pYB‐Dual vector described here. First, a large library of enzyme variants can be created and transformed into the Rosetta 2 (DE3) pLysS strain. A promiscuous/weak pre‐screen in the bacteria can then be carried out, selecting for enzyme variants that still retain function, which would remove most enzyme variants with deleterious mutations. This sifted bacterial library can then be extracted and transformed into the yeast for a more thorough and in‐depth enzymatic screen. The strength of the pYB‐Dual vector is that there is no need to pre‐select a handful of best‐preforming enzyme variants. All of the best‐performing enzyme variants can be extracted from *E. coli* and then immediately transformed into yeast. Thus, providing virtually no bottlenecks for the identification of enzymes with new functions. Also, as only functional enzyme variants would be collected from the bacterial host and transformed into the yeast, the theoretical maximum size of the yeast library would be considerably smaller than for the initial bacterial transformed library, thus facilitating a faster down‐stream screening by for example fluorescence‐activated cell sorting (FACS). This strategy should increase the probability of finding an optimal enzyme variant for the yeast cell factory. Currently, this protein engineering strategy would require a sub‐cloning step that takes time and increases the risk of losing sequence diversity within the library. With the pYB‐Dual expression vector no sub‐cloning step is required, and a pre‐screened bacterial library can immediately be transferred into the yeast for large‐scale protein purification. This would undoubtedly save time while accelerating both innovation and subsequent validation.

## 5 CONCLUSION

Producing high‐value peptides, proteins, and medicines in microbial cell‐factories is an attractive proposition to uncouple the biotech industry from complex and fragile supply‐chains that sometimes involve multiple countries on many different continents.^14^ Local production of complex compounds that otherwise cannot be sourced close by have the power to solve many potential bottlenecks within the biotech industry. Two important cell‐factory platforms that could fill this niche are *E. coli* and *S. cerevisiae*. However, transitioning protein and enzymes from one organism to another is not always an easy proposition, especially if those organisms do not belong to the same domain of life, that is, pro‐ versus eukaryote. Here, we have designed a dual‐expression vector that can facilitate high‐titer expression in both organisms. By also employing a mitigation strategy involving the overexpression of rare tRNA molecules within the bacterial host we can easily overcome a codon‐usage barrier, which allows us to focus enterally on optimizing the codon‐usage of our gene‐of‐interest for our end‐host; *S. cerevisiae*. We believe that this strategy will facilitate an easy and straightforward workflow that will enable further exploration and innovation of both yeast and bacterial cell‐factories going forward.

## AUTHOR CONTRIBUTIONS

**Marcus Wäneskog:** Conceptualization; data curation; formal analysis; methodology; investigation; funding acquisition; writing – original draft; visualization; writing – review and editing. **Trine Bertram Rasmussen:** Investigation. **Emil D. Jensen:** Writing – review and editing; project administration; supervision; funding acquisition; resources.

## CONFLICT OF INTEREST STATEMENT

No conflicts of interest.

### PEER REVIEW

The peer review history for this article is available at https://www.webofscience.com/api/gateway/wos/peer‐review/10.1002/btpr.3482.

## Supporting information


**Data S1** Supporting Information.

## Data Availability

The data that support the findings of this study are available from the corresponding authors, Marcus Wäneskog and Emil D. Jensen, upon request.

## References

[1] Baeshen NA , Baeshen MN , Sheikh A , et al. Cell factories for insulin production. Microb Cell Fact. 2014;13(1):141. doi:10.1186/s12934-014-0141-0 25270715 PMC4203937

[2] Porro D , Sauer M , Branduardi P , Mattanovich D . Recombinant protein production in yeasts. Mol Biotechnol. 2005;31(3):245‐260. doi:10.1385/MB:31:3:245 16230775

[3] Nilsson J , Jonasson P , Samuelsson E , Stahl S , Uhlén M . Integrated production of human insulin and its C‐peptide. J Biotechnol. 1996;48(3):241‐250. doi:10.1016/0168-1656(96)01514-3 8862001

[4] Yuan J , Mo Q , Fan C . New set of yeast vectors for shuttle expression in *Escherichia coli* . ACS Omega. 2021;6(10):7175‐7180. doi:10.1021/acsomega.1c00339 33748631 PMC7970545

[5] Saida F , Uzan M , Lallemand JY , Bontems F . New system for positive selection of recombinant plasmids and dual expression in yeast and bacteria based on the restriction ribonuclease RegB. Biotechnol Prog. 2003;19(3):727‐733. doi:10.1021/bp0257224 12790631

[6] Kozak M . Point mutations define a sequence flanking the AUG initiator codon that modulates translation by eukaryotic ribosomes. Cell. 1986;44(2):283‐292. doi:10.1016/0092-8674(86)90762-2 3943125

[7] Dvir S , Velten L , Sharon E , et al. Deciphering the rules by which 5′‐UTR sequences affect protein expression in yeast. Proc Natl Acad Sci U S A. 2013;110(30):2792‐2801. doi:10.1073/pnas.1222534110 23832786 PMC3725075

[8] Shaner NC , Lambert GG , Chammas A , et al. A bright monomeric green fluorescent protein derived from *Branchiostoma lanceolatum* . Nat Methods. 2013;10(5):407‐409. doi:10.1038/nmeth.2413 23524392 PMC3811051

[9] Daniel E , Onwukwe GU , Wierenga RK , Quaggin SE , Vainio SJ , Krause M . ATGme: open‐source web application for rare codon identification and custom DNA sequence optimization. BMC Bioinform. 2015;16(1):303. doi:10.1186/s12859-015-0743-5 PMC4578782 26391121

[10] Shine J , Dalgarno L . The 3′‐terminal sequence of *Escherichia coli* 16S ribosomal RNA: complementarity to nonsense triplets and ribosome binding sites. Proc Natl Acad Sci U S A. 1974;71(4):1342‐1346. doi:10.1073/pnas.71.4.1342 4598299 PMC388224

[11] Chen YJ , Liu P , Nielsen AAK , et al. Characterization of 582 natural and synthetic terminators and quantification of their design constraints. Nat Methods. 2013;10(7):659‐664. doi:10.1038/nmeth.2515 23727987

[12] Sikorski RS , Hieter P . A system of shuttle vectors and yeast host strains designed for efficient manipulation of DNA in *Saccharomyces cerevisiae* . Genetics. 1989;122(1):19‐27. doi:10.1093/genetics/122.1.19 2659436 PMC1203683

[13] Dower WJ , Miller JF , Ragsdale CW . High efficiency transformation of *E. coli* by high voltage electroporation. Nucleic Acids Res. 1988;16(13):6127‐6145. doi:10.1093/nar/16.13.6127 3041370 PMC336852

[14] Tucker EL , Cao Y , Fox ER , Sweet BV . The drug shortage era: a scoping review of the literature 2001–2019. Clin Pharmacol Ther. 2020;108(6):1150‐1155. doi:10.1002/cpt.1934 32521038

