# Supplementary material for: A strategy for successful dual‐species protein expression of genes with non‐optimal codon usage destined for bacterial and yeast cell factories
Source: Biotechnol Prog. 2024 May 17;40(6):e3482. doi: 10.1002/btpr.3482 (PMC11659799; doi:10.1002/btpr.3482)
Supplement: Supplementary file 1 — Data S1 Supporting Information. [file BTPR-40-e3482-s001.docx]

**Supplementary material for:**

**A strategy for successful dual-species protein expression of genes with non-optimal codon usage destined for bacterial and yeast cell factories**

Marcus Wäneskog*, Trine Bertram Rasmussen, Emil D. Jensen*

Novo Nordisk Foundation Center for Biosustainability, Technical University of Denmark, DK-2800 Kgs. Lyngby, Denmark

* To whom correspondence should be addressed. Marcus Wäneskog, e-mail: marcusw@biosustain.dtu.dk or Emil D. Jensen, e-mail: emdaje@biosustain.dtu.dk

**This supplementary file includes:**

Supplementary methods

Supplementary tables S1-S3

Supplementary figures S1-S2

**Construction of the pYB-Dual(mNG) vector**

The previously described vector pEDJ333 (Addgene ID: 177272) was used as template for the construction of the pYB-Dual vector. This vector is a pRS415 based yeast shuttle vector ^1^ that contains a GAL1 promoter, a Cas9 CDS and a CYC1 terminator ^2^. The pEDJ333 vector was first linearized by cutting the Cas9 CDS with FD SmaI (Thermo Fisher Scientific). A linear DNA fragment containing the mNeonGreen(mNG) CDS was synthesized and codon-optimized by Thermo Fisher Scientific GeneStrings, then PCR amplified with oligos; 176 and 177, to provide the pT7-RNAP promotor sequence and homology to the pEDJ333 vector. Both the mNG and the pRS415 vector fragments were then transformed into MW23, using the lithium acetate ssDNA heat-shock method ^3^, to allow for homologues recombination (gap-repair). Yeast colonies that were leucine prototrophs were PCR screened using oligos; 62 and 202, to identify positive recombination events, and then verified by sequencing, using the same oligos.

**Construction of the pYB-Dual(Empty) vector**

The mNG CDS in the pYB-Dual(mNG) vector was removed by FD XbaI (Thermo Fisher Scientific) and FD BamHI (Thermo Fisher Scientific). The resulting linear DNA was then end-repaired using T7 DNA polymerase (New England Biolabs), followed by self-circularization, using T4 DNA ligase (Thermo Fisher Scientific). The resulting circular molecule was then transformed into MW76. This new vector molecule has the XbaI site destroyed and the BamHI site re-created. Ampicillin resistant bacterial colonies were PCR screened using oligos; 62 and 202, to identify positive cloning events, and then verified by sequencing, using the same oligos.

**Construction of the pRS415(mNG) vector**

The pYB-Dual(mNG) vector was linearized with FD XbaI (Thermo Fisher Scientific) and transformed into MW23, along with oligo; 318, to remove the pT7-RNAP and bacterial ribosome binding site sequence, through homologues recombination (gap-repair), using the lithium acetate ssDNA heat-shock method ^3^. Yeast colonies that were leucine prototrophs were PCR screened using oligos; 202 and 208, to identify positive recombination events, and then verified by sequencing, using the same oligos.

**Construction of the pUC19(mNG) vector**

The pUC19 backbone along with an ampicillin resistance marker (identical to the pYB-Dual vector), and a CcdB counterselection marker, was PCR amplified with oligos; 142 and 290, from a related pESC-ccdB vector ^4^. The resulting linear DNA was self-circularized, using T4 DNA ligase (Thermo Fisher Scientific), and then transformed into a CcdB resistant *E. coli* strain (Thermo Fisher Scientific). The vector DNA from several ampicillin resistant bacterial colonies were extracted and then verified by sequencing, using oligo; 319. This new pUC19-AmpR-CatR-CcdB vector was then further PCR amplified with oligos; 319 and 325, removing the ccdB and CatR genes. The pT7-RNAP-SD-mNG fragment from pYB-Dual was PCR amplified with oligos; 169 and 208. Both fragments were then blunt-end ligated together, by T4 DNA ligase (Thermo Fisher Scientific), and then transformed into MW166. The vector DNA from surviving (lacking the ccdB gene), ampicillin resistant bacterial colonies, that also were chloramphenicol sensitive, were extracted and then verified by sequencing, using oligo; 142 and 208.

**Table S1. Bacterial strains used in this study.**

| **Strain number** | **Genotype** | **Origin** |
| --- | --- | --- |
| MW76 | BL21(DE3) *F- ompT hsdSB (rB-mB-) gal dcm /pLysS CamR* | New England Biolabs |
| MW79 | BL21(DE3) *F- ompT hsdSB (rB-mB-) gal dcm /pLysS /pYB-Dual(CEN6-ARS4)(pGAL1-pT7-tCYC1) Leu+ AmpR CamR* | This Study |
| MW80 | BL21(DE3) *F- ompT hsdSB (rB-mB-) gal dcm /pLysS /pYB-Dual(CEN6-ARS4)(pGAL1-pT7-SD-YTE-mNG^yeast-opt^-tCYC1) Leu+ AmpR CamR* | This Study |
| MW81 | BL21(DE3) *F- ompT hsdSB (rB-mB-) gal dcm* | This Study |
| MW150 | TOP10 *F– mcrA Δ(mrr-hsdRMS-mcrBC) φ80lacZΔM15 ΔlacX74 recA1 araD139 Δ(ara-leu)7697 galU galK λ–rpsL(StrR) endA1 nupG* | Thermo Fisher Scientific |
| MW166 | Rosetta 2 (DE3) *F- ompT hsdSB(rB- mB-) gal dcm (DE3) /pLysSRARE2 CamR* | Sigma-Aldrich |
| MW169 | BL21(DE3) *F- ompT hsdSB (rB-mB-) gal dcm /pYB-Dual(CEN6-ARS4)(pGAL1-pT7-tCYC1) Leu+ AmpR* | This Study |
| MW170 | BL21(DE3) *F- ompT hsdSB (rB-mB-) gal dcm /pYB-Dual(CEN6-ARS4)(pGAL1-pT7-SD-YTE-mNG^yeast-opt^-tCYC1) Leu+ AmpR* | This Study |
| MW171 | Rosetta 2 (DE3) *F- ompT hsdSB(rB- mB-) gal dcm (DE3) /pLysSRARE2 /pYB-Dual(CEN6-ARS4)(pGAL1-pT7-tCYC1) Leu+ AmpR CamR* | This Study |
| MW172 | Rosetta 2 (DE3) *F- ompT hsdSB(rB- mB-) gal dcm (DE3) /pLysSRARE2 /pYB-Dual(CEN6-ARS4)(pGAL1-pT7-SD-YTE-mNG^yeast-opt^-tCYC1) Leu+ AmpR CamR* | This Study |
| MW176 | Rosetta 2 (DE3) *F- ompT hsdSB(rB- mB-) gal dcm (DE3) /pLysSRARE2 /pUC19(pT7-SD-YTE-mNG^yeast-opt^) AmpR CamR* | This Study |

**Table S2. Yeast strains used in this study.**

| **Strain number** | **Genotype** | **Origin** |
| --- | --- | --- |
| MW23 | CEN.PK2-1C *MATa ura3-52 his3∆1 leu2-3,112 trp1-289 MAL2-8c SUC2* | Lab Collection |
| MW142 | CEN.PK2-1C *MATa ura3-52 his3∆1 leu2-3,112 trp1-289 MAL2-8c SUC2 /pYB-Dual(CEN6-ARS4)(pGAL1-pT7-tCYC1) Leu+ AmpR CamR* | This Study |
| MW143 | CEN.PK2-1C *MATa ura3-52 his3∆1 leu2-3,112 trp1-289 MAL2-8c SUC2 /pYB-Dual(CEN6-ARS4)(pGAL1-pT7-SD-YTE-mNG^yeast-opt^-tCYC1) Leu+ AmpR CamR* | This Study |
| MW177 | CEN.PK2-1C *MATa ura3-52 his3∆1 leu2-3,112 trp1-289 MAL2-8c SUC2 /pRS415(CEN6-ARS4)(pGAL1-YTE-mNG^yeast-opt^-tCYC1) Leu+ AmpR CamR* | This Study |

**Table S3. Oligos used in this study.**

| **Name** | **Sequence 5’-3’** |
| --- | --- |
| 62 | CAGGCTTTACACTTTATGCTTCC |
| 142 | GCAGCCTACTCGCTATTGTC |
| 169 | 5’Phosfate-TAATACGACTCACTATAGGTCTAGAGAAAGAGGGGAGAATTAAAAATGGTGTCTAAAGGTGAAGAGGACAAC |
| 176 | AATATACCTCTATACTTTAACGTCAAGGAGAAAAAACTATAATCTGTCATTAATACGACTCACTATAGGTCTAG |
| 177 | CAGGTTGTCTAACTCCTTCCTTTTCGGTTAGAGCGGATGAATGCACGCGATGGATCCTTATTTGTATAATTCATCC |
| 202 | CTAATACTTTCAACATTTTCG |
| 208 | 5’Phosfate-TGAATGCACGCGATGGATCCTTA |
| 290 | 5’Phosfate-GGAAATGTGCGCGGAACC |
| 318 | AATATACCTCTATACTTTAACGTCAAGGAGAAAAAACTATAATCTGTCATAATTAAAAATGGTGTCTAAAGGTGAAGAGGACAACATGGCTTCTTTGCCA |
| 319 | GAAGCCGAACGACTTGTAAGAG |
| 325 | TCACTGACTCGCTGCGCTCG |

**Figure S1. Sequence of pYB-Dual(Empty) vector.**
Color coded as followed: pGAL1, pT7-RNAP, BamHI, tCYC1

CCCTCGAGAGCGACCTCATGCTATACCTGAGAAAGCAACCTGACCTACAGGAAAGAGTTACTCAAGAATAAGAATTTTCGTTTTAAAACCTAAGAGTCACTTTAAAATTTGTATACACTTATTTTTTTTATAACTTATTTAATAATAAAAATCATAAATCATAAGAAATTCGCTTATTTAGAAGTGTCAACAACGTATCTACCAACGGAATGCGTGCGATGAGCCCCATTATCTTAGCCTAAAAAAACCTTCTCTTTGGAACTTTCAGTAATACGCTTAACTGCTCATTGCTATATTGAAGTACGGATTAGAAGCCGCCGAGCGGGCGACAGCCCTCCGACGGAAGACTCTCCTCCGTGCGTCCTGGTCTTCACCGGTCGCGTTCCTGAAACGCAGATGTGCCTCGCGCCGCACTGCTCCGAACAATAAAGATTCTACAATACTAGCTTTTATGGTTATGAAGAGGAAAAATTGGCAGTAACCTGGCCCCACAAACCTTCAAATCAACGAATCAAATTAACAACCATAGGATAATAATGCGATTAGTTTTTTAGCCTTATTTCTGGGGTAATTAATCAGCGAAGCGATGATTTTTGATCTATTAACAGATATATAAATGCAAAAGCTGCATAACCACTTTAACTAATACTTTCAACATTTTCGGTTTGTATTACTTCTTATTCAAATGTCATAAAAGTATCAACAAAAAATTGTTAATATACCTCTATACTTTAACGTCAAGGAGAAAAAACTATAATCTGTCATTAATACGACTCACTATAGGTCTAGGATCCATCGCGTGCATTCATCCGCTCTAACCGAAAAGGAAGGAGTTAGACAACCTGAAGTCTAGGTCCCTATTTATTTTTTTATAGTTATGTTAGTATTAAGAACGTTATTTATATTTCAAATTTTTCTTTTTTTTCTGTACAGACGCGTGTACGCATGTAACATTATACTGAAAACCTTGCTTGAGAAGAGCTCCAGCTTTTGTTCCCTTTAGTGAGGGTTAATTGCGCGCTTGGCGTAATCATGGTCATAGCTGTTTCCTGTGTGAAATTGTTATCCGCTCACAATTCCACACAACATAGGAGCCGGAAGCATAAAGTGTAAAGCCTG

**Figure S2. Sequence of pYB-Dual(mNG^yeast-opt^) vector.**
Color coded as followed: pGAL1, pT7-RNAP, XbaI, Bacterial Shine-Dalgarno and Yeast Translation-Enhancing Sequence, mNG^yeast-opt^, BamHI, tCYC1

CCCTCGAGAGCGACCTCATGCTATACCTGAGAAAGCAACCTGACCTACAGGAAAGAGTTACTCAAGAATAAGAATTTTCGTTTTAAAACCTAAGAGTCACTTTAAAATTTGTATACACTTATTTTTTTTATAACTTATTTAATAATAAAAATCATAAATCATAAGAAATTCGCTTATTTAGAAGTGTCAACAACGTATCTACCAACGGAATGCGTGCGATGAGCCCCATTATCTTAGCCTAAAAAAACCTTCTCTTTGGAACTTTCAGTAATACGCTTAACTGCTCATTGCTATATTGAAGTACGGATTAGAAGCCGCCGAGCGGGCGACAGCCCTCCGACGGAAGACTCTCCTCCGTGCGTCCTGGTCTTCACCGGTCGCGTTCCTGAAACGCAGATGTGCCTCGCGCCGCACTGCTCCGAACAATAAAGATTCTACAATACTAGCTTTTATGGTTATGAAGAGGAAAAATTGGCAGTAACCTGGCCCCACAAACCTTCAAATCAACGAATCAAATTAACAACCATAGGATAATAATGCGATTAGTTTTTTAGCCTTATTTCTGGGGTAATTAATCAGCGAAGCGATGATTTTTGATCTATTAACAGATATATAAATGCAAAAGCTGCATAACCACTTTAACTAATACTTTCAACATTTTCGGTTTGTATTACTTCTTATTCAAATGTCATAAAAGTATCAACAAAAAATTGTTAATATACCTCTATACTTTAACGTCAAGGAGAAAAAACTATAATCTGTCATTAATACGACTCACTATAGGTCTAGAGAAAGAGGGGAGAATTAAAAATGGTGTCTAAAGGTGAAGAGGACAACATGGCTTCTTTGCCAGCTACTCATGAATTGCATATTTTCGGTTCCATCAACGGTGTTGATTTCGATATGGTTGGTCAAGGTACTGGTAATCCAAATGATGGTTACGAAGAGTTGAACCTGAAATCTACAAAAGGTGACTTGCAATTCTCCCCATGGATTTTGGTTCCACATATTGGTTACGGTTTCCACCAATATTTGCCATATCCAGATGGTATGTCTCCATTTCAAGCTGCTATGGTTGATGGTTCTGGTTACCAAGTTCATAGAACCATGCAATTTGAAGATGGTGCTTCTTTGACCGTCAACTACAGATATACTTACGAAGGTTCCCATATTAAGGGTGAAGCTCAAGTAAAAGGTACAGGTTTTCCAGCTGATGGTCCAGTTATGACTAATTCTTTGACTGCTGCTGATTGGTGCAGATCCAAAAAGACTTACCCAAACGATAAGACCATCATCTCTACTTTCAAGTGGTCTTACACTACTGGTAACGGTAAGAGATATAGATCTACTGCTAGAACTACTTACACCTTCGCTAAACCTATGGCTGCTAACTACTTGAAGAATCAACCTATGTACGTGTTCAGAAAGACCGAATTGAAACACTCCAAAACCGAGTTGAACTTCAAAGAATGGCAAAAGGCTTTCACCGATGTTATGGGTATGGATGAATTATACAAATAAGGATCCATCGCGTGCATTCATCCGCTCTAACCGAAAAGGAAGGAGTTAGACAACCTGAAGTCTAGGTCCCTATTTATTTTTTTATAGTTATGTTAGTATTAAGAACGTTATTTATATTTCAAATTTTTCTTTTTTTTCTGTACAGACGCGTGTACGCATGTAACATTATACTGAAAACCTTGCTTGAGAAGAGCTCCAGCTTTTGTTCCCTTTAGTGAGGGTTAATTGCGCGCTTGGCGTAATCATGGTCATAGCTGTTTCCTGTGTGAAATTGTTATCCGCTCACAATTCCACACAACATAGGAGCCGGAAGCATAAAGTGTAAAGCCTG

**References**

1. Sikorski RS, Hieter P. A system of shuttle vectors and yeast host strains designed for efficient manipulation of DNA in Saccharomyces cerevisiae. *Genetics*. 1989;122(1):19-27. doi:10.1093/genetics/122.1.19

2. Jensen ED, Laloux M, Lehka BJ, Pedersen LE, Jakočiūnas T, Jensen MK, Keasling JD. A synthetic RNA-mediated evolution system in yeast. *Nucleic Acids Res*. 2021;49(15):e88-e88. doi:10.1093/nar/gkab472

3. Gietz RD, Schiestl RH. High-efficiency yeast transformation using the LiAc/SS carrier DNA/PEG method. *Nat Protoc*. 2007;2(1):31-34. doi:10.1038/nprot.2007.13

4. Jensen NB, Strucko T, Kildegaard KR, David F, Maury J, Mortensen UH, Forster J, Nielsen J, Borodina I. EasyClone: method for iterative chromosomal integration of multiple genes in Saccharomyces cerevisiae. *FEMS Yeast Res*. 2014;14(2):238-248. doi:10.1111/1567-1364.12118
